# Georgia Medical Association

**Published:** 1884-05-20

**Authors:** 


### *GEORGIA MEDICAL ASSOCIATION.*

At the late meeting of the Georgia Medical Association, Dr. Jno. Thad. Johnson, of the Committee on nominations, reported the following medical gentlemen as the chairmen of committees in the several districts, to-wit:

1st District—Section on Practice, W. Duncan, M.D.
" " " " Gynecology, R. J. Nunn, M.D.
2d District—Section on Practice, T. S. Dekel, M.D.
" " " " Surgery, A. P. Taylor, M.D.
" " " " Gynecology, T. S. Hopkins, M.D.
3d District—Section on Practice, S. B. Hawkins, M.D.
" " " " Surgery, R. H. Pate, M.D.
" " " " Gynecology, T. F. Walker, M.D.
4th District—Sectiou on Practice, C. D. Smith, M.D.
" " " " Surgery, W. A. Pool, M.D.
" " " " Gynecology, W. W. Fitts, M.D.
5th District—Section on Practice, W. D. Bizzell, M.D.
" " " " Surgery, W. P. Nicolson, M.D.
" " " " Gynecology, J. G. Earnest, M D.
6th District—Section on Practice, L. B. Alexander, M.D.
" " " " Surgery, T. H. Kenan, M.D.
" " " " Gynecology, T O. Powell, M.D.
7th District—Section on Practice, W. S. Kendrick, M.D.
" " " " Surgery, W. D. Wells, M.D.
" " " " Gynecology, Robert Battey, M.D.
8th District—Section on Practice, J Gerdine, M.D.
" " " " Surgery, G. W. Milligan, M.D.
" " " " Gynecology, J. E. Pope, M.D.
9th District—Section on Practice, J. W. Bailey, M.D.
" " " " Surgery, W. J. Rusk, M.D.
" " " " Gynecology, L. G. Hardeman, M.D.
10th District—Section on Practice, S. D. Brantley, M.D.
" " " " Surgery, A. G. Whitehead, M.D,
" " " " Gynecology, H. F. Campbell, M.D.

